# Deep Learning Enables Accurate Prediction of Interplay Between lncRNA and Disease

**DOI:** 10.3389/fgene.2019.00937

**Published:** 2019-10-09

**Authors:** Jialu Hu, Yiqun Gao, Jing Li, Xuequn Shang

**Affiliations:** ^1^School of Computer Science, Northwestern Polytechnical University, Xi’an, China; ^2^Centre for Multidisciplinary Convergence Computing, School of Computer Science, Northwestern Polytechnical University, Xi’an, China; ^3^Key Laboratory of Big Data Storage and Management, Northwestern Polytechnical University, Xi’an, China; ^4^Ming De College, Northwestern Polytechnical University, Xi’an, China

**Keywords:** lncRNA, neural network, large dataset, non-linear, computational model

## Abstract

Many studies have suggested that lncRNAs are involved in distinct and diverse biological processes. The mutation of lncRNAs plays a major role in a wide range of diseases. A comprehensive information of lncRNA-disease associations would improve our understanding of the underlying molecular mechanism that can explain the development of disease. However, the discovery of the relationship between lncRNA and disease in biological experiment is costly and time-consuming. Although many computational algorithms have been proposed in the last decade, there still exists much room to improve because of diverse computational limitations. In this paper, we proposed a deep-learning framework, NNLDA, to predict potential lncRNA-disease associations. We compared it with other two widely-used algorithms on a network with 205,959 interactions between 19,166 lncRNAs and 529 diseases. Results show that NNLDA outperforms other existing algorithm in the prediction of lncRNA-disease association. Additionally, NNLDA can be easily applied to large-scale datasets using the technique of mini-batch stochastic gradient descent. To our best knowledge, NNLDA is the first algorithm that uses deep neural networks to predict lncRNA-disease association. The source code of NNLDA can be freely accessed at https://github.com/gao793583308/NNLDA.

## Introduction

There are about 30,000–40,000 protein-coding genes in the human genome, which are only about twice as many as in worm or fly (Lander et al., 2001). But the majority of the human genome transcripts are non-coding RNAs, in particular, long non-coding RNAs (lncRNAs) (Geng et al., 2013). Protein-coding genes account for only 1.5% of the human genome. However, researchers observed a total of 62.1% and 74.7% of the human genome to be covered by either processed or primary transcripts respectively (Djebali et al., 2012). This suggests that lncRNA also plays an important role in biological processes. Recent studies revealed that numerous sets of non-coding RNA involved in distinct and diverse biological processes, such as cell proliferation, RNA binding complexes, immune surveillance, ESC pluripotency, neuronal processes, morphogenesis, gametogenesis, and muscle development (Mitchell et al., 2009). Furthermore, some important lncRNA biomarkers were found in a wide range of human diseases. For example, the expression of HOTAIR would induce androgen-independent (AR) activation, which plays a central role in establishing an oncogenic cascade that drives prostate cancer progression. It can also drive AR-mediated transcriptional programs in the absence of androgen (Zhang et al., 2015). So finding the relationship between lncRNA and disease can not only help us understand the mechanism of disease, but also accelerate the discovery of biomarker. However, discovering the potential relationship between lncRNA and disease by experimental ways are costly and time-consuming. Thus, many computational models have been proposed to predict potential connection patterns by utilizing existing data such as LncRNADisease (Geng et al., 2013), LncRNAdb (Cheng et al., 2015), and NONE-CODE (Cheng et al., 2015).

The existing computational models can be divided into two categories. The first class of methods make predictions based on the similarity of artificial definitions. It assumed that similar diseases or lncRNA have similar connection patterns. Take a simple example, if we know that disease(i) is related to lncRNA(i) and disease(i) and disease(j) are very similar. It’s obvious that we can infer that disease(j) and lncRNA(i) are also related. This algorithm needs to collect a lot of additional data to accurately define similarity. If the definition of similarity is accurate, the algorithm can achieve high performance. For example, LncRDNetFlow utilizes a flow propagation algorithm to integrate multiple networks based on a variety of biological information including lncRNA similarity, protein-protein interactions, disease similarity, and the associations between them to infer lncRNA-disease associations (Zhang et al., 2017a). IRWRLDA construct lncRNA expression similarity and lncRNA functional similarity to make prediction (Chen et al., 2016). RWRlncD infer potential human lncRNAdisease associations by implementing the random walk with restart method on a lncRNA functional similarity network (Sun et al., 2014). BiWalkLDA integrating interaction profiles and gene ontology information to construct similarity network. Such an algorithm also has KATZLGO (Zhang et al., 2017b) and IDHI-MIRW (Fan et al., 2019). It can be seen that this algorithm first constructs the similarity network based on the relevant data and then making prediction according to the constructed similarity. The second class of methods make predictions based on matrix factorization (MF). Their core idea is to learn a similarity rather than artificial definition similarity. This actually turns the prediction process into a classification question. For each lncRNA and disease, the aim of MF is to learn a latent factor to represent them and then make prediction based on learned latent factors. In this way, no additional knowledge is needed to define similarity. This method is widely used in prediction lncRNA-disease association. For example, the algorithm of MFLDA decomposes data matrices of heterogeneous data sources into low-rank matrices *via* matrix tri-factorization to explore and exploit their intrinsic and shared structure (Fu et al., 2017). SIMCLDA models the lncRNA-disease association prediction problem as a recommendation task and solves it with inductive matrix completion (IMC) (Lu et al., 2018).

The known lncRNA-disease association data used by current algorithms is derived from LncRNADisease (Geng et al., 2013). This database was proposed in 2013 and does not contain much lncRNA and disease (almost 300 lncRNA and 700 diseases). Because the data is relatively small, even though the existing prediction algorithms can achieve high accuracy, many results are repetitive and therefore cannot provide more valuable results. Fortunately, recently, a larger dataset LncRNADisease 2.0 can be used (Bao et al., 2019). LncRNADisease 2.0 curated 19,166 lncRNAs, 823 circRNAs, and 529 diseases from 3878 literatures. Although the form of data remains unchanged, only the increase in the amount of data makes previous algorithms not applicable to LncRNADisease 2.0. For methods that need to artificially define similarity, it is difficult to collect the additional information needed comprehensively in the face of such large data. So, it is difficult to define an appropriate similarity for prediction. For the method based on MF, the time cost of the algorithm is unacceptable with the increase of data. Besides, MF is actually a linear model of latent factors, so it cannot describe more complex relational patterns well (He et al., 2017). As we all know, deep learning can be applied to large-scale data and learn complex non-linear relationships by means of mini-batch stochastic gradient descentand and nonlinear activation function. In recent years, deep neural networks have yielded immense success on object detection (Ren et al., 2017), recommendation System (Zhou et al., 2017), single cell denoising (Eraslan and Simon, 2019; Peng et al., 2019), and many other fields. However, no deep learning-based algorithm has been proposed to predict potential lncRNA-disease association. In this article, we will introduce our proposed framework NNLDA which uses neural networks to predict lncRNA-disease association. To our best knowledge, NNLDA is the first algorithm that uses deep neural networks to predict lncRNA-disease association. Experiments show that NNLDA can be well applied to large data and to learn more complex non-linear relationships.

## Method

Our prediction framework NNLDA is improved based on the MF method. In this section, I will first introduce the method of MF and point out its shortcomings. Then, we will explain how we solve these shortcomings and introduce the procedure of NNLDA in detail.

### Matrix Factorization (MF)

MF is a frequently used method in the problem of predicting lncRNA-disease association (Fu et al., 2017; Lu et al., 2018). Its core idea is to learn a corresponding latent factor for each lncRNA and disease. The dot product of the latent factor was used to represent the possible score of corresponding lncRNA and disease. Take the prediction of lncRNA-disease association, for example. First, we should construct an adjacency matrix *A_nl×nd_*, where *n_l_* is the number of lncRNA and *n_d_* is the number of diseases. *A_ij_* = 1 represents that the *i^th^* lncRNA is associated with *d_j_*, otherwise, *A_ij_* = 0. Then, we assign a k-dimensional latent factor L(i) for each lncRNA(i) and a k-dimensional latent factor D(i) for each disease(i). These latent factors are usually randomly initialized at the beginning and then be adjusted by some optimization algorithm such as stochastic gradient descent. Now, we can use the dot product of the latent factor to re-estimate A. For each pair of lncRNA(i) and disease(j), we predict its association using ${\hat{A}}_{ij}=\sum_{n=1}^{k}L_{in}D_{nj}$. Our objective funcition is to minimize the following loss function:

$$Loss=\sum_{i=1}^{n_{l}}\sum_{j=1}^{n_{d}}\left(A_{ij}-{\hat{A}}_{ij}\right)$$

A new L and D can be learned by minimizing loss. This loss is actually equivalent to $Loss=\;||A-LD|{|}_{F}^{2}$, which was frequently used in other literatures because the dot product of vectors can be seen as the angle of vectors in space (*a*⋅*b* = |*a*||*b*|*cos*(*a*,*b*)). So, matrix factorization actually maps each lncRNA and disease into k-dimensional space and then defines the relationship between lncRNA and disease by using the length and angle of the latent factor. However, there are several shortcomings in doing so: (1) There are limitations in utilizing the angle between latent factor to define the relationship between lncRNA and disease. Take two-dimensional space as an example, suppose we now learn three latent factors: *a*_1_(1,0), *a*_2_(0,1), *a*_3_(1,1), if we also have latent factor *a*_4_ and we want the angle between *a*_4_ and *a*_1_, *a*_2_ to be as small as possible, but the angle between *a*_2_ and *a*_3_ to be as large as possible. Obviously, no matter where *a*_4_ is, it can’t be satisfied. Of course, we can describe this relationship by adding spatial dimensions, but the increase of k actually increases the risk of over-fitting. It can be concluded that angle can’t actually describe some complex relationship patterns perfectly. (2) The time complexity of matrix decomposition is too high. When calculating the loss, it needs to calculate all possible connections between lncRNA and disease. As the amount of data increases, the time required is unacceptable. Besides directly optimizing, global loss is easy to fall into local minima.

### Making Matrix Factorization Applicable to Large Data

In order to make the matrix factorization method suitable for large-scale data, we made some improvements to the original method and implemented the method with tensorflow. We named this method NNMF, which is different from the traditional MF method in two aspects:

1. Unlike previous MF, full data is used to minimize loss. We adopt mini-batch stochastic gradient descent to train model. This means that we use only one batch data per round to minimize loss, which makes our algorithm suitable for large-scale data.
2. The traditional matrix factorization uses mean square error or absolute value error to measure loss. Its goal is to $min||A-LD|{|}_{F}^{2}$. In NNMF, we use cross-entropy as our loss function, which is proved to be more applicable to classification problems and easier to optimize.

**Figure 1:**
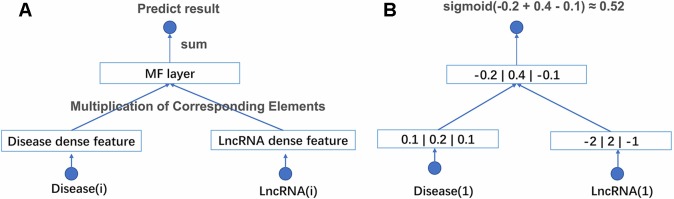
**(A)** The structure of NNMF. Each lncRNA and disease is projected into a k-dimensional space. It means each lncRNA and disease would be represented by a corresponding k*1 eigenvector. The relationship between lncRNA and disease is measured by the dot product of their corresponding eigenvector. The activation function is sigmoid. **(B)** A toy example of the NNMF, where k is set to 3.

With above two improvements, NNMF can be adapted to large-scale data. The structure of the network and an example of computational processes are shown in **Figure 1**. NNMF takes lncRNA(i) and disease(j) as its input and outputs the probability of the relationship between lncRNA(i) and disease(j). First, the network generates a dense latent factor for corresponding lncRNA(i) and disease(j). This operation is done by embedding lookup function in tensorflow. Then, the corresponding position elements of the two vectors are multiplied and summed. Sigmoid activation functions are added to limit output to between 0 and 1. With the predicted results, we can calculate the cross-entropy loss to adjust the corresponding latent factor. To avoid storing the whole data set into memory each time we take a batch data to train, the batch size is set to 1,024. This process is repeated until the loss is no longer reduced. NNMF changes the way of training and the loss function compared with the traditional matrix decomposition algorithm. With these small changes, NNMF can be adapted to large-scale data easily.

### Learning More Complex Relationships by Using Full Connectivity Layer

Matrix factorization actually maps lncRNA and disease into k-dimensional space, and then measures their relationship by using dot product of latent factors. This approach undoubtedly has its limitations. In order to learn more complex non-linear features, a natural idea is to use the full connection layer of the neural network to improve it. Similar to the NNMF process, we initialize a latent factor for each lncRNA and disease at the beginning. Then, we concatenated the latent factors and add full connection layers to learn more complex relationships. RelU activation function is used on each full connection layer to increase the non-linear description ability of the network. Sigmoid activation functions are added to limit output to between 0 and 1. Considering that using full connection layer alone may increase the risk of over-fitting. We adopt the following two strategies to prevent over-fitting:

1. Add L2 regularization to latent factors and full connection layer to limit models from learning too complex features.
2. The deep part is trained together with NNMF. In this way, we cannot only learn more diverse connection relationships, but also improve the generalization ability of the model.

**Figure 2:**
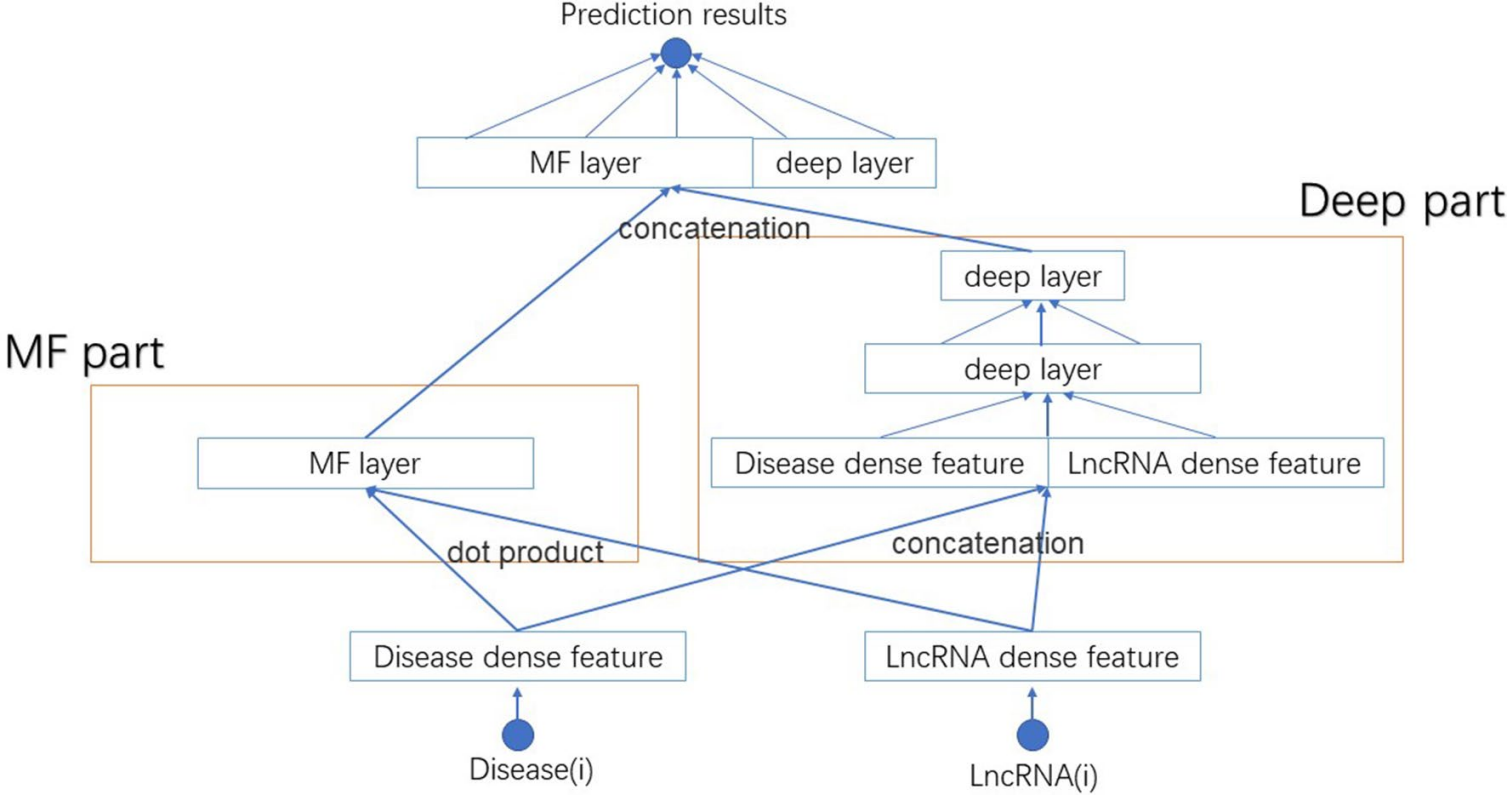
The structure of NNLDA. MF part is same as NNMF. Deep part use several full connection layers to learn complex association relationships. Their results are concatenated together to make final predictions.

We name this new model NNLDA. It means predicting lncRNA-disease association by means of neural networks. The overall structure of NNLDA is shown in **Figure 2**. First, for each lncRNA and disease, we will find their corresponding latent factors. MF part multiplies the corresponding elements of latent factors and deep part use several full-connection layers to learn the complex relationship between lncRNA and disease. Their results are concatenated together and connected to a full connection layer for final prediction. Sigmoid activation function is added to limit output to between 0 and 1. NNLDA learns more complex relational patterns by combining dot product of latent factors and full connectivity layer. Because NNLDA uses mini-batch stochastic gradient descent to minimize loss, it can also be well applied to large-scale data. We believe that NNLDA can perfectly solve the shortcomings of traditional MF methods.

### Implementation

NNLDA is implemented in Python 3.5 and uses TensorFlow1.12.0. Length of latent factor is set to 32. Three full-connection layers with lengths of 32, 16 and 8 are added in deep part. L2 regulation is added in all full-connection layers and latent factors to prevent over-fitting and regulation rate is set to 0.01. We use adam for optimization with learning rate 0.01. Epoch is set to 100 and batch size is set to 1024.

## Experiment

### Dataset

Unlike previous algorithms which usually perform on small data sets such as LncRNADisease database, we use LncRNADisease 2.0 to measure the results of the algorithm. LncRNADisease 2.0 shows that there exists 205,959 interactions between 19,166 lncRNAs and 529 diseases. We believe that more valuable results can be found by using larger data. Such large-scale data also challenges previous algorithms. The experimental data can be downloaded from http://www.rnanut.net/lncrnadisease/. We remove all repeating records with the same lncRNA and disease, and all these non-human associations. Finally, we retained 187,55 lncRNA and 463 disease with 177,899 associations.

### 10-Fold Cross Validation

To test the algorithm performance, we employed a widely-used strategy, 10-fold cross validation. Known lncRNA and disease associations are divided into 10 copies. In each round, nine of them are used to train algorithms and the remaining one is used as a test set. Notice that we need negative samples to train the algorithm, but in fact we don’t know which lncRNAs are not associated with diseases. So, for each known LncRNA-disease, we will randomly sample four lncRNA that do not interact by this disease as negative samples. When predicting test sets, we no longer use AUC as an evaluation criterion. This is because AUC needs to compute all possible associations. This means that if there are n lncRNA and m disease, we need to calculate n*m possible cases and then generate a rank list. It’s obvious that it’s unrealistic when the data set is large. So we adopt a new evaluation strategy. For each test sample, we will sample 99 random lncRNA that not interact by this disease. The model scores 99 negative samples and one positive sample to generate the corresponding rank list. Then, we use Hit Ratio (HR) to assessment results. The HR intuitively measures whether the test item is present on the top-k rank list and we can interpret HR (K) as the probability of positive samples appearing in top-k rank list. If the test sample is in the first k of rank list, its value is plus one. The hit rate value can be obtained by dividing the final hit value by the number of test samples. The higher the hit rate, the higher the likelihood that true sample will appear in the top-k rank list.

### The Effects of Parameters

#### Length of Latent Factors

**Figure 3:**
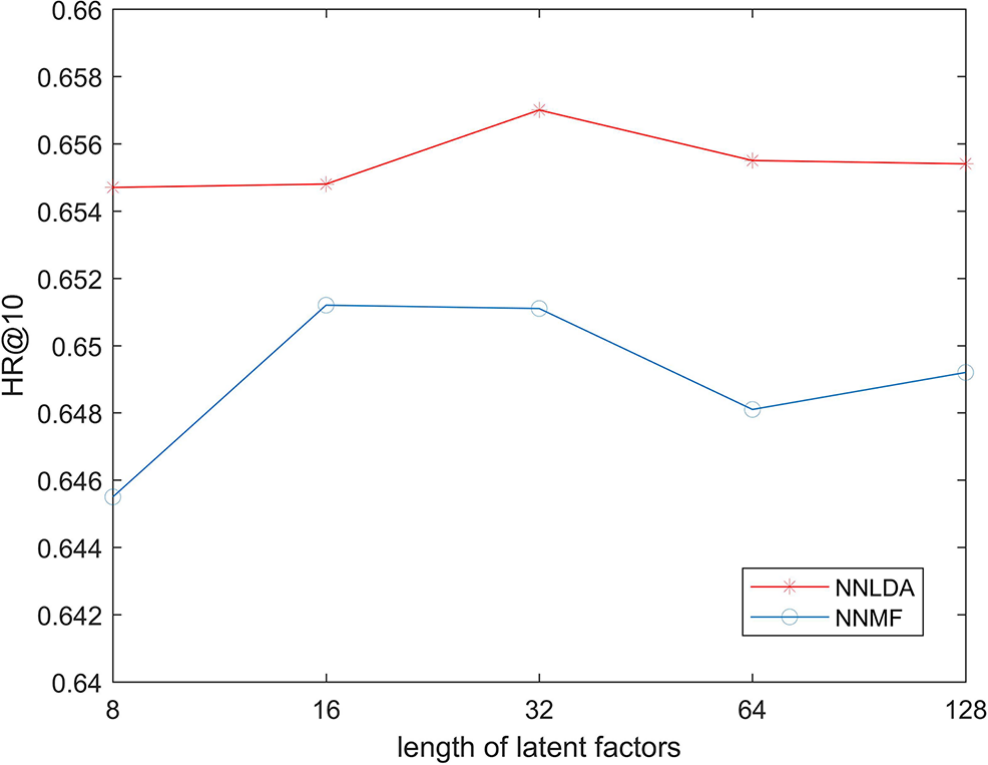
HR @ k Three Algorithms under Different value of k.

In the first step of NNMF and NNLDA, both lncRNA and disease need to be mapped into a k-dimensional vector. This vector is called latent factors. Here, k is an artificially defined parameter and represents the dimension of feature space. If the value of k is very small, the model cannot learn complex relationships. If the value of k is big, the risk of over-fitting of the model increases. In order to test possible effects on the performance of the algorithm under different value of k, we changed the value of k in 8, 16, 32, 64, and 128 each time, and then calculated the HR 10. Because KNN does not use latent factors, we only compared NNMF and NNLDA here. The experimental results show in **Figure 3**. The result shows that the length of latent factors don’t actually have much impact on the hit ration. This is because we added L2 regularization to latent factors. Even if the length of latent factors increases, it will not be over-fitting data. If no regularization is added, the loss of the model decreases rapidly and over-fitting will occur soon.

#### Number of Layers

**Figure 4:**
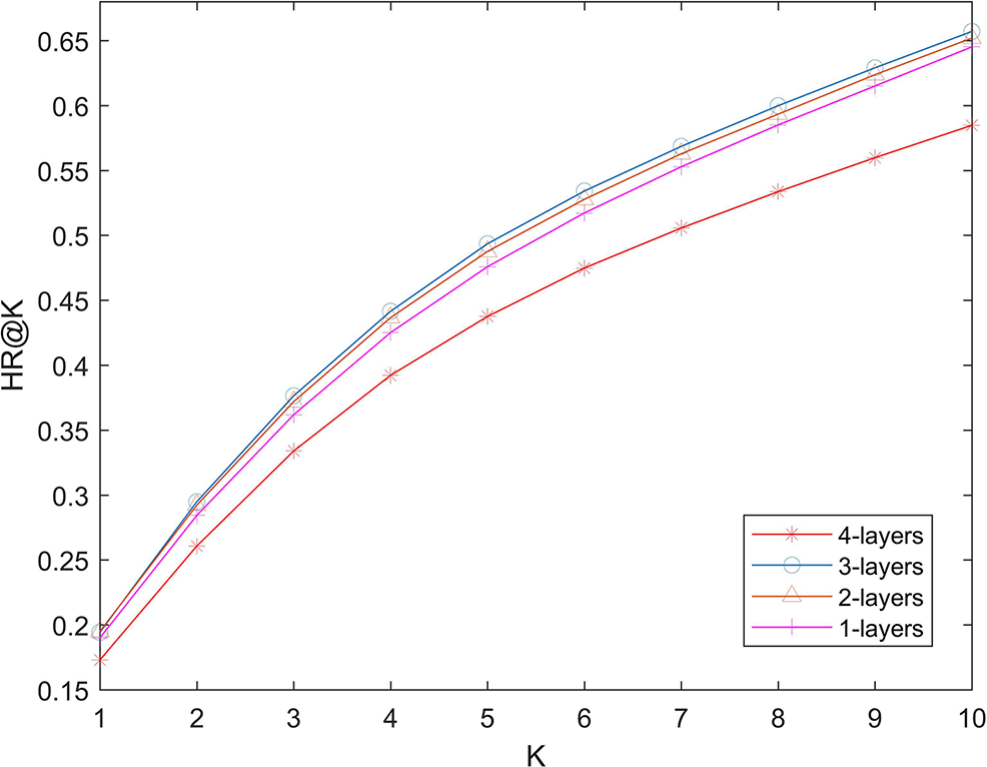
Effects of lengths of latent factors.

We used several full-connection layers in deep part to learn more complex relationships. More layers can theoretically learn more complex models, which also increases the risk of over-fitting. In order to test the possible effect of number of layers on the performance of the algorithm. We changed the number of layers in 1-layer (32), two-layer (32 and 16), three-layer (32, 16, and 8) and four-layer (32, 16, 8, and 4), and calculate the hit ration value. The experimental results are shown in **Figure 4**. It can be seen that increasing the number of layers of the network will not greatly improve the effectiveness of the algorithm. Algorithm performance is poor when the number of layers is 4. This shows that even if we use L2 regularization to prevent over-fitting, the number of layers of the network should not be too big.

### Comparison With Other Algorithms

Because we use LncRNADisease 2.0 to compare the performance of our algorithm. Traditional algorithms cannot be applied to such large dataset. So, although many computational models have been proposed, they cannot be used for comparison. We have made some changes to the traditional algorithm. NNMF can be seen as a matrix factorization algorithm suitable for large-scale data. For algorithms that need to define similarity artificially, we implement an algorithm manually based on the idea of KNN. The specific process is as follows: First, we calculate the gauss similarity between diseases which is widely used in other papers. Then for each disease, we will find 40 diseases that are most similar to it and use their average interaction profile to make predictions.

**Figure 5:**
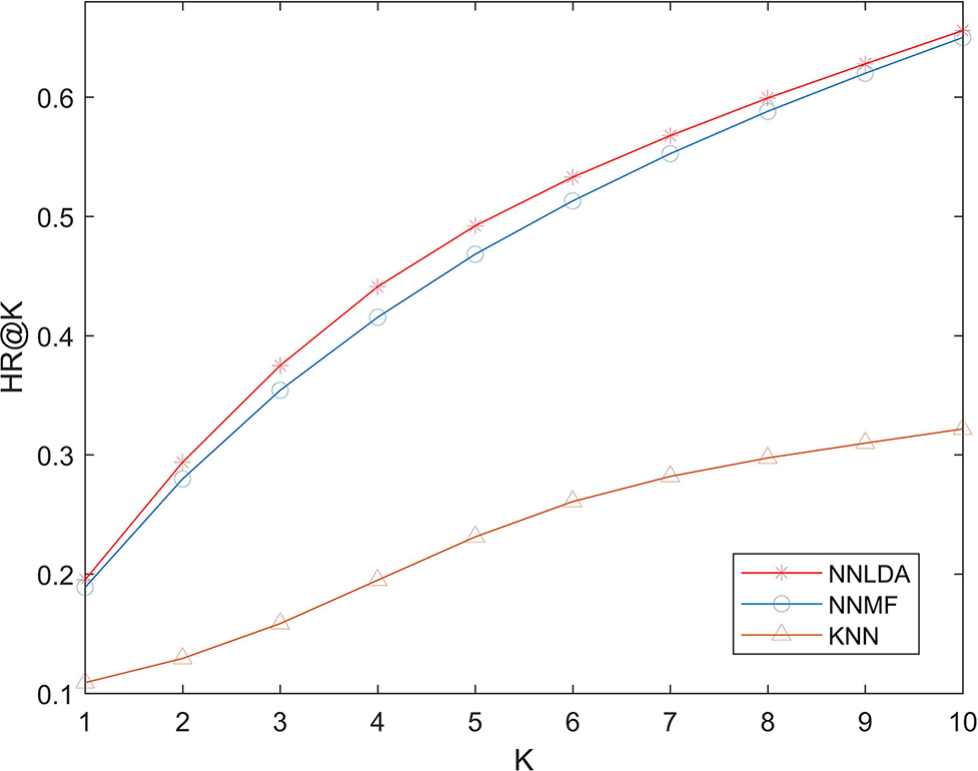
Effects of lengths of latent factors.

We compare NNLDA with other two computational methods (NNMF and KNN) of lncRNA-disease association prediction in terms of HR. All algorithms use the same data to make predictions. The experimental results are shown in **Figure 5**. It can be seen that the performance of KNN is very poor. This is because similarity-based algorithms need to artificially define the similarity between diseases and then make predictions based on similarity. As the amount of data increases, additional data becomes more and more difficult to obtain. Because of this, it is difficult to define an accurate and reasonable similarity. So, the performance of this algorithm is limited by similarity. Comparing NNLDA and NNMF, we can find that NNLDA outperforms NNMF in all k values. In fact, NNLDA can be seen as model fusion of NNMF and full connectivity layer. This shows that more complex connection relationships can be learned by using the full-connection layer.

## Conclusion

Many recent studies suggest that lncRNAs are strongly associated with various complex human diseases. Therefore, the discovery of the potential association between lncRNA and diseases helps to understand the biological processes and underlying mechanisms of diseases. Many prediction algorithms have been proposed to predict lncRNA-disease association. Although the algorithm can achieve high accuracy, traditional prediction algorithms can no longer be applied to more and more large-scale data. In this paper, we propose NNLDA to predict lncRNA-disease association. NNLDA uses mini-batch stochastic gradient descent and cross-entropy loss to enable the algorithm to be applied to large-data sets and use full-connection layer to make up for the deficiency of MF expression ability. Our contributions can be summarized as follows: 1) NNLDA is the first algorithm can predict lncRNA-disease association on large datasets. 2) NNLDA is the first algorithm to use neural network to predict potential lncRNA-disease association. Compared with traditional MF algorithm, NNMF can better describe their relationship by using full-connection layer. In the experimental part, we compare NNLDA, KNN, and NNMF. The experimental results show that NNLDA performs better in terms of hit rate on LncRNADisease 2.0 database. The experiment of parameter influence shows that NNLDA is robust to different parameter setting.

## Data Availability Statement

All datasets generated/analyzed for this study are included in the manuscript/supplementary files.

## Author Contributions

JH designed the computational framework, JH and YG implemented the program. YG and JL performed all the analyses of the data and wrote the manuscript. XS is the major coordinator, who contributed a lot of time and efforts in the discussion of this project. All authors read and approved the final manuscript.

## Funding

Publication costs were funded by the National Natural Science Foundation of China (Grant No. 61702420). This project has also been funded by the National Natural Science Foundation of China (Grant No. 61332014, 61702420 and 61772426); the China Postdoctoral Science Foundation (Grant No. 2017M613203); the Natural Science Foundation of Shaanxi Province (Grant No. 2017JQ6037); the Fundamental Research Funds for the Central Universities (Grant No. 3102018zy032); and the Top International University Visiting Program for Outstanding Young Scholars of Northwestern Polytechnical University.

## Conflict of Interest

The authors declare that the research was conducted in the absence of any commercial or financial relationships that could be construed as a potential conflict of interest.
